# Stratigraphic signatures of mass extinctions: ecological and sedimentary determinants

**DOI:** 10.1098/rspb.2018.1191

**Published:** 2018-09-12

**Authors:** Rafał Nawrot, Daniele Scarponi, Michele Azzarone, Troy A. Dexter, Kristopher M. Kusnerik, Jacalyn M. Wittmer, Alessandro Amorosi, Michał Kowalewski

**Affiliations:** 1 Florida Museum of Natural History, University of Florida, 1659 Museum Road, Gainesville, FL 32611, USA; 2 Dipartimento di Scienze Biologiche, Geologiche e Ambientali, University of Bologna, Via Zamboni 67, 40126 Bologna, Italy; 3 Gerace Research Centre, University of the Bahamas, San Salvador, Bahamas; 4 Department of Geological Sciences, State University of New York at Geneseo, One College Circle, Geneseo, NY 14454, USA

**Keywords:** mass extinction, sequence stratigraphy, Signor–Lipps effect, sampling bias, stratigraphic palaeobiology, Holocene

## Abstract

Stratigraphic patterns of last occurrences (LOs) of fossil taxa potentially fingerprint mass extinctions and delineate rates and geometries of those events. Although empirical studies of mass extinctions recognize that random sampling causes LOs to occur earlier than the time of extinction (Signor–Lipps effect), sequence stratigraphic controls on the position of LOs are rarely considered. By tracing stratigraphic ranges of extant mollusc species preserved in the Holocene succession of the Po coastal plain (Italy), we demonstrated that, if mass extinction took place today, complex but entirely false extinction patterns would be recorded regionally due to shifts in local community composition and non-random variation in the abundance of skeletal remains, both controlled by relative sea-level changes. Consequently, rather than following an apparent gradual pattern expected from the Signor–Lipps effect, LOs concentrated within intervals of stratigraphic condensation and strong facies shifts mimicking sudden extinction pulses. Methods assuming uniform recovery potential of fossils falsely supported stepwise extinction patterns among studied species and systematically underestimated their stratigraphic ranges. Such effects of stratigraphic architecture, co-produced by ecological, sedimentary and taphonomic processes, can easily confound interpretations of the timing, duration and selectivity of mass extinction events. Our results highlight the necessity of accounting for palaeoenvironmental and sequence stratigraphic context when inferring extinction dynamics from the fossil record.

## 1. Introduction

Stratigraphic distributions of last occurrences (LOs) of fossil taxa in sedimentary successions have been used to quantify onsets, durations and intensities of mass extinctions (e.g. [1–4]), track geographical and environmental variation in extinction rates [4–6], and relate those rates to concurrent changes in geochemical and sedimentological proxies [2,4,5]. This type of palaeontological data has been thus crucial for high-resolution reconstructions of the temporal and ecological dynamics of mass extinction events and identification of their extrinsic drivers. Nevertheless, the observed stratigraphic position of the LO of a species typically predates its actual time of extinction [7]. In addition, as demonstrated by Signor & Lipps [8], artificial truncation of the observed stratigraphic range of a taxon can simply arise from a random sampling process, which can make abrupt extinction events appear gradual in the fossil record. To correct for this ‘Signor–Lipps effect’ [9], the timing and pattern of extinctions have been commonly estimated from stratigraphic data by assuming that fossil occurrences are essentially random and controlled primarily by sampling [7].

However, the focus on random sampling effects alone does not fully account for the complexity of the geological record and non-random nature of stratigraphic patterns [10]. Most outcrop- and core-based extinction studies implicitly assume that the positions of LOs logged at a given location provide an accurate chronology of extirpation or extinction events, especially so, if corrected for the Signor–Lipps effect. However, the local distribution of LOs is not only affected by sampling intensity, but also by systematic changes in facies and sediment accumulation rates that occur in response to regional or global drivers such as tectonic subsidence and eustatic change [11]. Consequently, a local stratigraphic expression of temporal variation in extinction rates is superimposed on ecological responses to lateral shifts of habitats through time, manifested in geological sections as vertical changes in composition and diversity of fossil assemblages. These changes can appear as sudden pulses in faunal turnover due to abrupt facies shifts, as well as due to non-deposition, stratigraphic condensation and erosion, often coincident with facies boundaries.

Numerical simulations that integrate eco-evolutionary and sequence stratigraphic models support those predictions: eco-stratigraphic processes can produce clusters of LOs concentrated at specific stratigraphic horizons such as flooding surfaces, surfaces of forced regression and sequence boundaries [10,12–15]. Such surfaces are typically associated with depositional hiatuses, stratigraphic condensation and non-Waltherian facies shifts [11,16]. Moreover, depositional architecture influences the stratigraphic distribution, taphonomic characteristics and temporal resolution of fossil concentrations [17–20], thus constraining the quantity and quality of palaeontological data retrievable from any given horizon.

The sequence stratigraphic control on fossil occurrences is worrisome because the fossil record of many prominent extinction events coincides with major sequence stratigraphic surfaces, suggesting that interpretations of these events may be partly, or even entirely, confounded by the stratigraphic architecture [10,21–23]. On the other hand, because trends in biodiversity and stratigraphic architecture may both be driven by common causative processes such as sea-level and climatic changes [24], it is difficult to distinguish clusters of LOs caused by elevated extinction rates from those generated by stratigraphic processes [10]. To avoid these uncertainties, we employ here the Holocene fossil record of living species to test the impact of the stratigraphic architecture on our ability to reconstruct the timing and tempo of past extinction events.

We evaluate how faithfully stratigraphic ranges of extant Adriatic molluscs are recorded in a series of cores that were drilled through a 40-metre-thick package of genetically related alluvial, coastal and shallow-marine strata of the Po Plain (northwest Italy). If the time when a given taxon was in existence was always accurately reflected by its stratigraphic range observed in local sections, LOs of extant species should coincide with the top of the succession. However, such a perfect record is implausible [8,25]. Here we test if the observed stratigraphic distribution of LOs can be explained by a random sampling alone (i.e. the Signor–Lipps effect), or is more consistent with combined effects of sampling and systematic variation in fossil occurrence rates as predicted by the sequence stratigraphic model. Our approach can also be directly linked to mass extinction studies by following a simple thought experiment [25]: imagine that a catastrophic event has just wiped out all shallow marine and brackish molluscan fauna of the Adriatic Sea. The extinction horizon would then correspond to the modern sedimentary surface preserved in the rock record by subsequent sedimentation. We ask if this hypothetical extinction event could be correctly reconstructed from cores drilled across the Po Plain. By tracing the stratigraphic occurrences of extant species with known ecological preferences, in the context of the established regional depositional and sequence stratigraphic frameworks [26], we can directly assess the imprint of facies control, stratigraphic architecture and sampling on the stratigraphic distribution of LOs.

## 2. Material and methods

### (a) Study area

The topmost 40-metre-thick succession of the Po coastal plain has been interpreted to represent the Last Glacial Maximum depositional sequence, which is currently accumulating in the Po Plain–Adriatic Sea system [27]. In the study area, the lowstand systems tract is primarily composed of fluvial and floodplain deposits of Late Pleistocene age. Overlying transgressive systems tract (TST) and highstand systems tract (HST) consist mainly of Holocene strata forming a transgressive–regressive, wedge-shaped succession, recently subdivided into a series of centennial units embedded within eight millennial-scale depositional units (parasequences in [26]; see ‘Geological setting’ in electronic supplementary material). The Holocene molluscan assemblages were studied in four cores for which environmental, sequence stratigraphic and chronostratigraphic interpretation have been previously established [19,20,26,28]. These cores form an L-shaped transect 35 km long that captures lateral variation in LO patterns along the depositional dip and strike (electronic supplementary material, figure S1). Fossiliferous deposits preserving estuarine, deltaic and marine assemblages occur up to the modern sedimentary surface in all four cores.

### (b) Dataset

A total of 229 bulk samples, each representing an approximately 10 cm core interval, were collected with an average vertical spacing of 0.57 m (range 0.05–1.52 m). The standardized volume of samples allows us to track changes in fossil abundance (i.e. the density of identifiable fossils per unit of a core). See Kowalewski *et al*. [29] for the details of sample processing. Specimens lacking species-level identification and all terrestrial or exclusively freshwater species were excluded, resulting in the dataset encompassing 119 species and 38 093 specimens (electronic supplementary material, dataset S1). Stratigraphic ranges of species observed in the cores were extended to the Late Pleistocene based on their occurrence in the underlying marine strata of the Last Interglacial (Marine Isotope Stage 5e [29]).

### (c) Estimating bathymetric preferences of species

We focused on water depth because it is one of the major factors controlling the distribution of marine benthic assemblages [15]. The independent estimates of the present-day bathymetric preferences of species were derived from the Italian Mollusc Census Database of the Italian National Agency for New Technologies, Energy and Sustainable Economic Development (ENEA; http://www.santateresa.enea.it/wwwste/malaco/home.htm), a compilation of benthic surveys along the Italian coast [30]. We only considered sampling stations located on the Adriatic Sea shelf. The restricted dataset consisted of 403 samples collected from 0 to 124 m water depth and containing approximately 240 000 individuals of 439 species in total.

The bathymetric preference of a species was estimated as the mean water depths across all sampling stations at which that species was found, weighted by the number of specimens at each of those stations. Because brackish habitats tend to be underrepresented in the ENEA database, this procedure may overestimate water depths of species reaching peak abundance in brackish conditions. The counts of both live and dead individuals were combined to assure that the calculated parameters reflect an environmental distribution of a species as would be observed in the fossil record. Preferred water depth was estimated for 72 out of 119 species (60.5%) recorded in the cores which were present at five or more sampling stations in the ENEA database.

### (d) Analytical methods

We tested whether the time and pattern of extinctions can be correctly estimated from the stratigraphic record under a simple model of a random distribution of fossil occurrences and constant sampling probability (uniform recovery potential) through time [7,31]. As recently noted by Wang & Marshall [7], this class of probabilistic methods continue to dominate palaeontological analyses, including the most recent studies.

First, we used empirically calibrated resampling simulations to assess if the stratigraphic patterns in LOs observed in the cores deviate significantly from the patterns that would be expected under uniform recovery potential of fossils. We designed two null models. The first model assumes a random distribution of species and uniform sampling intensity throughout the stratigraphic succession. The second model relaxes the latter condition by allowing the sample size to vary according to the trends in fossil abundance actually observed in the cores. See the electronic supplementary material for the details of the resampling procedures.

Second, we applied several methods of testing for abrupt versus stepwise or gradual extinction patterns: (i) a simple graphical approach based on the relationship between stratigraphic abundance (proportion of samples in which a given species was observed) and position of LO [25], (ii) a likelihood-ratio test for the simultaneous extinction of all species [32], and (iii) a two-step algorithm estimating the number of extinction pulses [33] (see the electronic supplementary material for details). We evaluated the ability of those methods to correctly identify the single simultaneous extinction event in each of the studied cores.

Finally, to test how accounting for systematic variation in fossil occurrence rates affects estimates of extinction times, we compared the performance of two methods for calculating confidence intervals (CIs) on the position of stratigraphic range endpoints. The classical method of Strauss & Sadler [34] assumes uniform recovery potential of fossils, while its generalization [35] allows recovery potential to vary with stratigraphic position according to a predefined recovery function. Following procedures of Holland [36], we estimated sample-level collection probabilities for each species using multivariate ordination (see the electronic supplementary material). For species with at least four occurrences in a given core, we calculated 50% and 95% CIs using both methods and compared the proportion of taxa for which the estimated range endpoint fell below the topmost sample in the core (the extinction horizon in our hypothetical scenario). All analyses were performed in R [37].

## 3. Results

**Figure 1.**
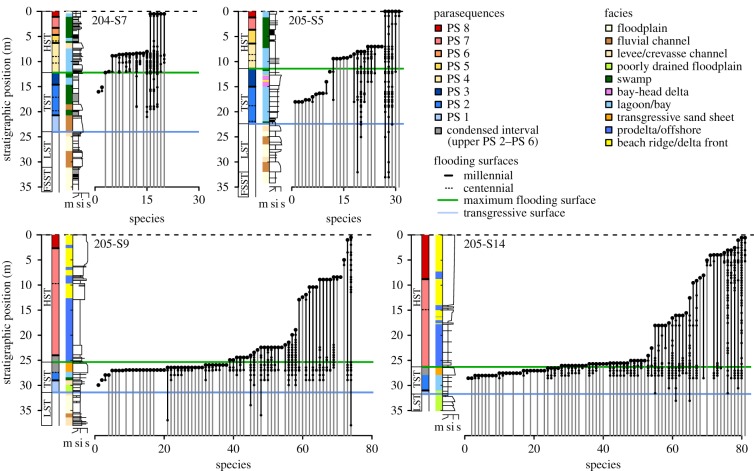
Stratigraphic ranges of extant mollusc species recorded in the proximal (cores 204-S7 and 205-S5, upper row) and distal (cores 205-S9 and 205-S14, lower row) parts of the Po coastal plain. Species are ordered according to their last occurrence below the hypothetical extinction horizon corresponding to the modern sediment surface (dashed horizontal line). Downward range extensions (marked in grey) are based on species occurrences in the underlying Late Pleistocene strata. Systems tracts, parasequences, facies associations and lithological logs are reported to the left of each range chart. Sequence stratigraphic interpretation follows Amorosi *et al.* [26]. FSST, falling stage systems tract; LST, lowstand systems tract; TST, transgressive systems tract, HST, highstand systems tract.

**Figure 2.**
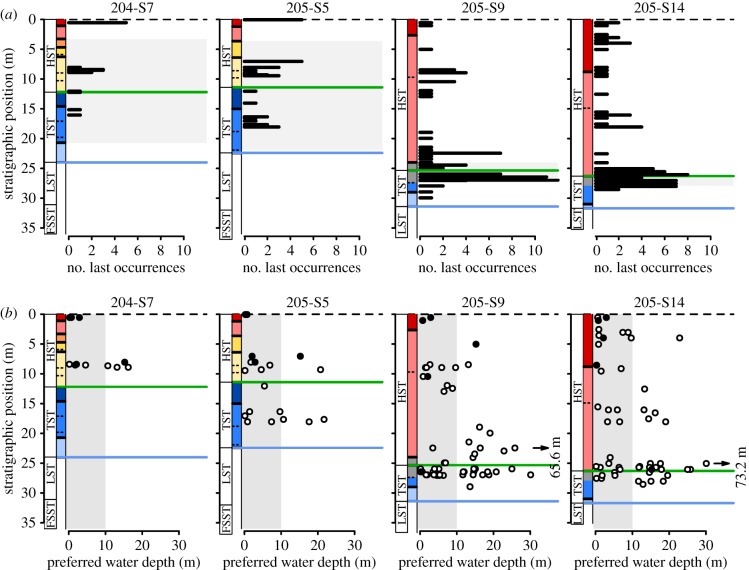
Clustering of LOs around the MFS (green line) associated with the disappearance of species preferring more offshore habitats. (*a*) The number of LOs observed at each sampled horizon. Grey rectangles delineate the stratigraphic interval isochronous with the strongly condensed portion of the two distal cores (the upper part of parasequence 2 to parasequence 6, see electronic supplementary material, figures S1 and S2). (*b*) Preferred water depth of mollusc species versus the stratigraphic position of their last occurrence. Water depths above the upper limit of the offshore transition zone (10 m) are marked in grey. Black points represent species reaching high abundance in brackish conditions. Arrows indicate outliers with much greater preferred water depth compared to the rest of the fauna. Note that several points representing different species can overlap. See figure 1 for the key to sequence stratigraphic units and parasequences.

Out of 119 extant species recorded in the cores, only six (5%) reach the top of the sedimentary succession (i.e. our hypothetical extinction horizon). Thus, taken at face value, the observed stratigraphic ranges would provide a strongly distorted record of the timing and pattern of extinctions (figure 1). Moreover, LOs do not follow a simple gradual pattern predicted by the Signor–Lipps effect [8,25]. Instead, LOs form distinct clusters, the number and stratigraphic position of which depend on the location of the core along the depositional profile (figures 1 and 2*a*).

In the two proximal cores, a major peak at 7–9.5 m core depth precedes the cluster of LOs corresponding to the hypothetical extinction horizon at the top of the cores (figures 1 and 2*a*). The older cluster occurs below the shift from lagoonal to swamp facies association and above a series of closely spaced centennial-scale flooding surfaces within the earliest HST (parasequence 4). In the core 205-S5, another cluster of LOs is located in the middle part of the TST (16–18 m; parasequence 2), which also consists of a set of centennial-scale units deposited in back-barrier settings. The same interval in core 204-S7, located further updip, records swamp and floodplain deposits mostly devoid of macrofossils (figure 1).

In the downdip cores, LOs are strongly clustered around the maximum flooding surface (MFS; figures 1 and 2*a*). This interval includes a metre-thick, highly fossiliferous transgressive sand sheet, recording a millennial-scale condensation, and, in its lower part, ecologically mixed (reworked) faunal assemblages [19,20]. The unit is capped by a thin veneer of offshore clays and silts recording the maximum water depths within the studied succession [26,38], followed by a thick package of sparsely fossiliferous prodelta muds. Above them, a smaller peak in LOs is located around the centennial-scale flooding surface within the HST (8.5–10.5 and 15.5–18 m in core 205-S9 and 205-S14, respectively; parasequence 7). This cluster precedes the onset of a progradation of the southern Po delta lobes (Spina and Volano) and deposition of coarser-grained delta front facies.

Combining fossil occurrences across the cores reduces the magnitude of range truncation: for 30 out of 64 species recorded in more than one core, the positions of their LOs shifted upward to a younger parasequence (electronic supplementary material, figure S2). However, even for data merged across all four cores, only 16 species (13%) reach the youngest parasequence 8, and 67 species (56%) do not range beyond the condensed interval around the MFS. Capturing the full extent of their stratigraphic ranges would require sampling of sections located even further downdip recording more offshore environments.

Given that all sampled species are still living in the region, truncations of their stratigraphic ranges must reflect incomplete sampling or change in the local habitat (i.e. facies shifts). Facies control on the stratigraphic distribution of LOs can be assessed using bathymetric preferences of individual species derived from independent surveys of recent benthic fauna. While shallow-water and brackish-tolerant species are recorded up to the very top of the cores, LOs of taxa preferring more offshore conditions are concentrated in the late TST and earliest HST (figure 2*b*). The disappearance of deeper-water species from the upper part of the sequence can be linked to the onset of a fast regional progradation during the HST (parasequences 7–8). In the two distal cores, this progradation led to deposition of a thick, shallowing-upward deltaic wedge [26] (electronic supplementary material, figure S1). The same shallowing trend is manifested in the two proximal cores by a reduction of marine influence in back-barrier settings.

**Figure 3.**
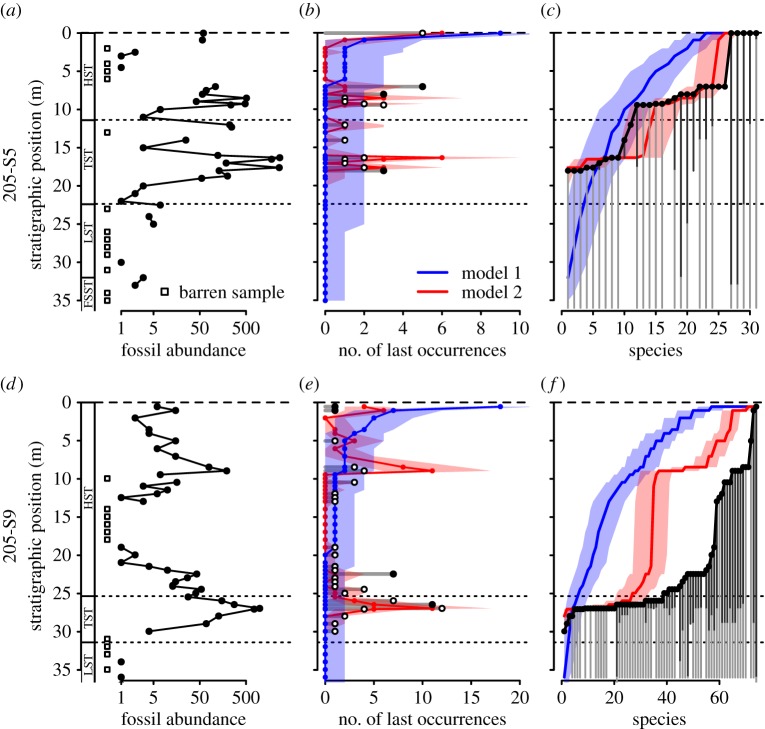
(*a*,*d*) Stratigraphic trends in fossil abundance (number of identifiable specimens per sample), and the results of two resampling models for (*b*,*e*) the expected number of LOs and (*c*,*f*) distribution of LOs (stratigraphic range endpoints) in cores (*a*–*c*) 205-S5 and (*d*–*f*) 205-S9. Model 1 assumes random distribution of species and uniform sampling. Model 2 also assumes random distribution of species but incorporates changes in fossil abundances observed throughout the cores. See the electronic supplementary material for details. Thick colour lines represent median values across 10 000 iterations and shading denotes 95% CIs. Filled black dots in (*b*) and (*e*) indicate the observed number of LOs that is significantly different from the number predicted for that stratigraphic position by either of the models. See figure 1 for the key to sequence stratigraphic units.

Systematic changes in fossil abundance represent another factor controlling the stratigraphic distribution of species. Because the volume of sediment sampled from each core interval was held constant, changes in shell density directly affect the number of specimens available for study and thus the likelihood of recording rare taxa. As a result, LOs of rare species should cluster in shell-rich horizons, where they are more likely to be sampled. Indeed, the position of LO clusters follows peaks in fossil abundance (figure 3*a,d*; electronic supplementary material, figures S3*a*,*d*).

The observed stratigraphic patterns in LOs depart significantly from the null model assuming both facies-independent distribution of species and uniform sampling intensity (model 1 in figure 3; electronic supplementary material, figure S3). Under those conditions, the probability of recording a given species depends only on its total abundance and remains constant throughout a core. In this model, the random truncation of stratigraphic ranges and differential sampling of rare versus common species lead to the classical Signor–Lipps pattern of downward smearing of LOs, stratigraphically manifested as a gradual decline in species diversity. By contrast, simulations incorporating vertical changes in fossil abundance always produce stepwise LOs patterns (model 2 in figure 3; electronic supplementary material, figure S3) and correctly predict the position and magnitude of many, though not all, LO clusters observed in the proximal cores. This suggests that some of the clusters of LOs can simply reflect a stratigraphic distribution of fossil-rich deposits. However, the distribution of LOs observed in the two distal cores is incongruent with the predictions of model 2. Variation in fossil abundance is thus insufficient to explain the position of LO clusters in these cores underscoring the importance of facies control on the distribution of LOs.

Model 1 in our simulations corresponds to the assumption of uniform preservation and recovery of fossils that underlies many probabilistic methods for estimating the actual time or pattern of extinctions from fossil occurrences [7,31]. Such methods do not account for sequence stratigraphic control on LO patterns. The relationship between stratigraphic abundance and position of LOs [25] suggests two extinction pulses (electronic supplementary material, figure S4). False stepwise extinction patterns are also favoured by the maximum-likelihood estimates for the number of extinction pulses [33] (electronic supplementary material, figure S5), while the likelihood-ratio test [32] incorrectly rejects the hypothesis of simultaneous extinction of all species in three out of the four cores (*p* = 0.17 for core 205-S5 and *p* < 0.001 for the remaining cores). Because all species are extant, the CIs on their stratigraphic range endpoints should extend to the modern sedimentary surface. However, the classical method [34] based on the assumption of random fossil occurrence, systematically underestimates the extent of species ranges (electronic supplementary material, figure S6). Incorporation of facies-dependent occurrence probabilities derived from multivariate ordination [35,36] (electronic supplementary material, figures S8 and S9) reduces the bias in the estimates of extinction time (electronic supplementary material, figure S7). While the classical 95% CIs do not reach the top of the succession for 44%, 55%, 74% and 79% of species in core 205-S5, 204-S7, 205-S14 and 205-S9, application of the ordination-based CIs reduces these numbers to 0%, 12%, 52% and 23% of species, respectively.

## 4. Discussion

Non-random truncation of stratigraphic ranges of extant species observed in the Holocene strata of the Po Plain reveals pervasive effects of stratigraphic architecture on the distribution of LOs preserved in the fossil record. LOs of taxa tend to concentrate within intervals of stratigraphic condensation and strong facies shifts associated with flooding surfaces in the TST and lower HST (figures 1 and 2*a*). Literal reading of these patterns would falsely suggest a multiphase extinction event: a regionally synchronous interval of highly elevated extinction rates around the time of maximum marine ingression followed by smaller extinction pulses (figure 2*a*). Moreover, the relationship between the stratigraphic position of LOs and ecological affinities of species (figure 2*b*), if considered without a reference to the vertical facies changes, could be mistaken as a signature of habitat-selective extinction: early extinction of offshore taxa followed by a more protracted, stepwise decline of shallow-water and brackish fauna.

Such sequence stratigraphic control on the distribution of LOs, easy to identify in the Holocene strata, can severely hamper interpretations of more ancient records, especially when outcrop area is restricted to a narrow portion of a depositional profile, temporal resolution is low, and ecology of extinct taxa poorly known [10,21,22]. Because the nature and intensity of stratigraphic controls depend on the location along a depositional profile, sequence architecture and tectonic setting [10,15,39], as well as on ecological and taphonomic characteristics of taxa [23,39–41], a variety of patterns in LOs can arise depending on the local context and taxonomic group under study. Indeed, contrasting interpretations of the number and timing of extinction pulses during the End-Permian mass extinction have been suggested to reflect different degrees to which sampling, facies control and stratigraphic incompleteness affect fossil occurrence patterns in individual sections [42].

The observed stratigraphic positions of LO clusters are consistent with the predictions of numerical simulations that modelled fossil occurrences as a function of bathymetry-dependent distribution of taxa and sequence stratigraphic architecture [10]. This agreement indicates that the effects of stratigraphic controls can be deduced from basic ecological and sequence stratigraphic principles, even when the models and empirical systems are only partly compatible. Whereas the numerical models simulated deposition over 10^6^–10^7^ years [10,12,14,39], the investigated succession represents only the last approximately 12 000 years. However, when considered in terms of the spatial extent, thickness of the strata, stratigraphic architecture and amplitude of the eustatic sea-level change, the studied succession is comparable to higher-order depositional sequences formed over much longer time scales. Consequently, the Quaternary fossil record of the Po Plain can serve as a good analogue for the field- or core-based mass extinction studies. Likewise, the discrepancy between the time interval covered by this study and typical species duration in molluscs (10^6^–10^7^ years) does not affect the results. Owing to its short temporal scale, the studied Holocene system is not affected by background extinctions, explicitly incorporated in the numerical models. Therefore, the observed clustering of LOs is produced solely by non-random truncation of stratigraphic ranges below a single mass extinction event (the modern sediment surface in our study). According to the models [10,12,13], the same types of sequence stratigraphic surfaces, at which these clusters were recorded, are expected to accumulate LOs of taxa even when extinction rate is constant through time. Consequently, the stratigraphic record of a mass extinction should be distorted even more severely in sedimentary successions spanning longer time scales, because LOs of the actual victims of a mass extinction would be clustered together with LOs of species that went extinct prior or after the event [10].

The stratigraphic distribution of species in Po Plain cores is controlled by both changes in depositional environments and vertical trends in fossil density. The first factor determines the composition of fossil assemblages that can be sampled at any given horizon, the second factor constrains the available sample size. Changes in environmental conditions and in fossil density are both driven by responses of sedimentary systems to relative sea-level changes and are thus closely correlated: stratigraphic intervals marked by strong facies shifts and faunal turnover represent horizons where net sedimentation rates are reduced and skeletal accumulations are more likely to form [17,18,20]. Clustering of LOs at these horizons results from the combined effects of (i) direct environmental control on species distribution and hard part production rates, and (ii) taphonomic and sedimentary processes underlying formation and preservation of fossil concentrations.

Shell-rich beds should on average capture a greater share of the regional species pool relative to shell-poor strata, even if regional diversity remains stable through time and sample size is held constant. This inflation in sample-level richness is expected due to effects of higher time-averaging [43,44], positive feedback between bioclast accumulation and local epibenthic diversity [45,46] and enhanced preservation potential of skeletal elements [47]. This interplay between facies control and sampling bias is illustrated by the prominent peak in LOs bracketing the MFS in the two distal cores (figure 2*a*). Owing to environmental condensation [19,20], LOs of both offshore and shallow-water species are clustered together in this interval (figure 2*b*). The disappearance of offshore species results from the subsequent shallowing, while the concentration of LOs of shallow-water forms reflects over-sampling of rare species due to high fossil densities.

Our null models suggest that even in a highly unlikely scenario of identical environmental preferences of all species, non-random stratigraphic distribution of skeletal concentrations can produce clusters of LOs that mimic sudden or stepwise extinction patterns (model 2 in figure 3). Thus, distinct patterns in LOs can result solely from changes in fossil abundance produced by stratigraphic variation in sedimentation rates, fossil preservation and habitat-dependent hard part production rates, all of which can be controlled by stratigraphic architecture. In other words, rather than being uniform and randomly distributed, the effects of sampling are closely tied to the stratigraphic architecture and covary with patterns caused by facies changes. More intensive collecting effort concentrated only around the postulated extinction horizon is thus unlikely to mitigate the problem.

In contrast to our results, Meldahl [25] observed the classic gradual pattern of LOs produced by the Signor–Lipps effect in cores taken from a tidal channel in Bahia la Choya, northern Gulf of California. However, the 70-cm-long cores used in that study captured a single facies association and remained fossiliferous throughout their length. Our numerical simulations (model 1 in figure 3) show that gradual backward smearing of LOs below the extinction horizon would occur only under assumptions of (i) continuous and uniform sampling, and (ii) facies-independent distribution of species or constant environment through time. Such ideal conditions, translating to uniform recovery potential of fossil, are unrealistic in sedimentary successions deposited over time scales relevant for mass extinction studies [11]. Yet methods based on this simplifying assumption are still widely used to infer timing of extinctions from stratigraphic data [7]. Such methods fail to correctly reconstruct the actual extinction pattern among investigated mollusc species (electronic supplementary material, figures S4–S6), demonstrating that accounting for random sampling alone is not enough to avoid the effects of stratigraphic controls on LOs.

Systematic changes in occurrence rates and sampling probabilities of fossil are the rule rather than exception [13,48]. As a result, reliance on statistical procedures that account for the incomplete fossil record by assuming a uniform recovery potential of fossils can lead to incorrect inferences on the timing and mode of mass extinction events. Methods that relax this unrealistic assumption (e.g. [49,50]) or incorporate independent information on facies-specific occurrence rates and/or sampling effort [35,36,51] may provide more reliable estimates of extinction times, as demonstrated by our results (electronic supplementary material, figure S7). However, datasets restricted to individual outcrops or local composite sections are unlikely to fully capture an environmental and stratigraphic distribution of a taxon (electronic supplementary material, figure S2), while extrapolation of locally observed patterns to regional or global scales is problematic [23]. Combining data from multiple sections spanning different basins and depositional environments may reduce the effects of facies and sampling [42]. Such analyses cannot rely on biostratigraphic correlations, as important index fossil groups are not immune to the effects of stratigraphic architecture [22,23].

Overcoming sequence stratigraphic controls on fossil occurrence, although challenging, is not impossible [15,23,52,53]. Several strategies for distinguishing extinction-generated pulses of LOs from those produced by stratigraphic architecture have been suggested [10]: (i) clusters of LOs not associated with sequence stratigraphic surfaces are likely to reflect elevated extinction rates; (ii) data from depositionally updip or downdip sections can verify if a locally observed LOs correspond to the actual extinctions rather than facies-related disappearances; and (iii) fossil assemblages from environmentally equivalent strata located below and above a postulated extinction pulse can be compared to test if the extinction rates across this interval exceed background extinction rates. Also, multivariate analyses can be used to compare the magnitude of faunal turnover across the postulated event to the variation in assemblage composition that is expected from changes in depositional environments or stacking patterns [23]. Novel quantitative approaches for estimating environmental affinity of fossil taxa [53,54] should further increase our ability to disentangle consequences of mass extinction events from ecological responses to lateral habitat migration.

## 5. Conclusion

Stratigraphic distribution of extant species demonstrates that interactions between ecological preferences of organisms and processes of sediment accumulation produce systematic changes in occurrence rates and sampling probabilities of taxa along a sedimentary succession. The resulting non-random truncation of stratigraphic ranges leads to clustering of LOs at specific sequence stratigraphic positions distorting the relative chronology of species extinctions. Such patterns can easily confound interpretations of the timing, duration and ecological selectivity of mass extinction events. Importantly, the effects of these eco-stratigraphic processes cannot be removed by methods that correct the Signor–Lipps effect under a model of uniform preservation and recovery of fossils.

Research strategies that account for the effects of stratigraphic architecture are data-intensive and rely on placing fossil occurrences in a rigorous palaeoenvironmental and sequence stratigraphic framework. They also typically require integration of data across multiple sections or sedimentary basin, thus often sacrificing temporal and spatial resolution. These challenges imposed by the nature of the stratigraphic record must be acknowledged and addressed before high-resolution reconstructions of past extinction dynamics are attempted. However, more conservative interpretations of the stratigraphic distribution of fossil taxa will maximize the accuracy of palaeobiological interpretations and reduce the risk of using false extinction patterns to formulate and test eco-evolutionary hypotheses.

## Data accessibility

The dataset and R code supporting this article are deposited at Dryad Digital Repository: http://dx.doi.org/10.5061/dryad.j9c15ff [55].
